# Impact of Nutritional Intervention on Taste Perception—A Scoping Review

**DOI:** 10.3390/foods10112747

**Published:** 2021-11-09

**Authors:** Alessandro Micarelli, Sandro Malacrida, Giacomo Strapazzon, Simona Mrakic-Sposta, Beatrice Micarelli, Nicolò Alessandrini, Valentina Carbini, Sara Caputo, Marika Falla, Marco Alessandrini

**Affiliations:** 1 Institute of Mountain Emergency Medicine, Eurac Research, 39100 Bolzano, Italy; sandro.malacrida@eurac.edu (S.M.); giacomo.strapazzon@eurac.edu (G.S.); marika.falla@unitn.it (M.F.); 2 ITER Center of Balance and Rehabilitation Research (ICBRR), UNITER ONLUS, 31010 Rome, Italy; beatricemicarelli@hotmail.it (B.M.); valentina.carbini@gmail.com (V.C.); 3 Institute of Clinical Physiology, National Research Council (CNR), 20162 Milan, Italy; simona.mrakicsposta@cnr.it; 4 Faculty of Philosophy, University of Rome Tor Vergata, 00133 Rome, Italy; dcapoccia@icloud.com; 5 L-Nutra Italia S.r.l., 20122 Milan, Italy; scaputo@l-nutra.com; 6 Center for Mind/Brain Sciences, CIMeC, University of Trento, 38068 Rovereto, Italy; 7 ENT Unit, Department of Clinical Sciences and Translational Medicine, University of Rome Tor Vergata, 00133 Rome, Italy; malessandrini63@gmail.com

**Keywords:** taste test, food, gustatory perception, diet, taste threshold

## Abstract

The aim of the present scoping review was to evaluate the impact of experimental meal loads or observational diet changes/habits on taste tests in both healthy subjects and patients. A systematic search performed in PubMed, Scopus, and Institute for Scientific Information (ISI) Web of Science electronic databases retrieved, respectively 2981, 6258, and 7555 articles from January 2000 to December 2020. A total of 17 articles were included for full-text review. Literature results were stratified according to the observational/interventional approach, the involvement of healthy subjects or patients, the taste test, and the meal/dietary changes. The present scoping review reinforced the notions postulating that certain taste tests (for example focusing on fatty acid, salt, or sugar) might be specifically influenced by the nutritional intervention and that other ones might be susceptible to a wide span of changes beyond the extent of tastant included in the specific food changes. This could also depend on the inhomogeneity of literature trend: The short duration of the intervention or the random type of meal load, unsuitability of the taste test chosen, and the presence of underlying disorders. Future studies for a better comprehension of taste tests reliability in relation to specific food changes are thus to be fostered.

## 1. Introduction

Taste is primarily a nutrient sensing system. The texture of the food and the rate at which it is eaten, have a profound effect on the time of orosensory exposure to foods and to satiation [1]. It has been established that slower eating leads to a longer exposure to taste [2,3,4,5]. Many studies suggest that the duration of the exposure to taste contributes significantly to the onset of satiation and the termination of a meal [6,7,8]. A longer duration of exposure to taste leads to earlier satiation [1]. A gut–brain signaling system has also been clearly demonstrated to influence taste responses to the consumption of food. The nucleus tractus solitarius (NTS) is the main entry point of the vagus nerve in the central nervous system, and thus, it receives afferent projections from most of the organs of the gastrointestinal (GI) tract [9]. In addition, the NTS also receives some cranial nerve afferents (i.e., facial and glossopharyngeal nerves) that convey extensive information on food texture, taste, smell, appearance, and palatability from the orosensory area, and conveys the ascending fibers ipsilaterally to the thalamus and insula, whereas some of them cross at the upper pons or midbrain level [10]. The gustatory system, therefore, has a predominately ipsilateral projection with some fibers crossing at the central level [10]. This system seems to respond rapidly to meal loads and to drive fast chemosensory-related reactions to the consumption of food [11]. On the other hand, chemosensory changes due to dietary habits are found to mainly rely on a wider network of areas including the hypothalamus and its sub-region responses, involved in the control of body energy homeostasis and its connections with NTS [11,12].

The comprehension of the reliability of testing taste—including both psychophysical and objective evaluations—appears of outmost relevance. Many clinical investigations have studied changes in the sense of taste in response to the meal load or to dietary habits as an effort to further cause changes along the pathways that support the extensive connections between taste and food intake. Within psychophysical evaluation techniques, there is a distinction between chemical (natural) (including the three-drop method [13,14], taste tablets and wafers [15,16], taste-strips [17,18,19], and the fatty acid threshold [20,21,22] or similar less-standardized methods [23,24,25]) and electrical testing tools (i.e., electrogustometry) [26,27]. The former techniques use basic taste solutions (sweet, sour, salty, bitter, and/or umami) to stimulate taste, and the latter techniques apply electrical currents to the surface of the tongue in order to elicit taste perceptions [20]. Both techniques suffer from the same shortcomings of psychophysical tests in general, which means that the patient must cooperate and must not be suffering from dementia. The main advantage of both tests is that they are quick and easy to perform [21] and have been used extensively in routine clinical practice and are currently accepted to be equally useful to measure gustatory function before and after any interventions [22]. Objective evaluation techniques—including gustatory event-related potentials [28,29], neuroimaging techniques (such as functional Magnetic Resonance Imaging [fMRI] and Positron Emission Tomography [PET]) [30,31,32], and the confocal microscopy [33,34]—are more difficult to perform [27] and require likewise patients’ cooperation.

The aim of the present scoping review was to evaluate the impact of experimental meal loads or observational diet changes/habits on taste tests in both healthy subjects and patients.

## 2. Scoping Review Methods

The scoping review and the meta-analysis were conducted following the guidelines of the Preferred Reporting Items for Systematic Review and Meta-Analysis (PRISMA) extension for scoping reviews (PRISMA-ScR) [35].

### 2.1. Eligibility Criteria

The literature search was performed to identify studies evaluating taste changes in response to a nutritional intervention in the last 20 years: meal or nutrient loads, diet and food consumption habits, and fasting periods. The inclusion criteria (Table 1) of the search strategy design are categorized according to the broad Population–Concept–Context (PCC) mnemonic recommended for scoping reviews [36,37].

The scoping review focuses on applications in experimental and clinical settings and on physiological research. Thus, studies on both adult healthy subjects and on patients were included. Studies were eligible only if psychophysical/objective, experimental/tailored objective taste tests were implemented after nutritional intervention, while studies investigating only behavioral, hedonic, and satiation aspects or mainly using questionnaires were excluded.

The search was restricted to observational and interventional studies only involving humans and published in English in peer-reviewed journals. Abstracts, conference proceedings and reports, retrospective studies, expert opinions, letters to the Editor, commentaries, case reports, and reviews were excluded.

### 2.2. Information Sources, Search Strategy, and Study Selection

A systematic search was performed in PubMed, Scopus, and ISI Web of Science electronic databases to identify primary references from January 2000 to December 2020. The following search string was used: (“meal” OR “food” OR “nutrition” OR “fasting” OR “hunger” OR “diet” OR “calory” OR “energy consumption” OR “consumption” OR “intake” OR “satiation” OR “feeding” OR “eating” OR “nourishment” OR “sustenance”) AND (“taste” OR “flavour” OR “gustatory” OR “test” OR “taste test” OR “taste perception” OR “gustatory perception” OR “taste sensitivity” OR “gustatory sensitivity” OR “taste strips” OR “chemosensory” OR “liking” OR “adverse reaction”). The database search was followed by a review of the citations from eligible studies. The studies were selected based on their title and their abstract using the online platform Rayyan [38]. The selected studies were read thoroughly to identify those suitable for inclusion in the scoping review.

### 2.3. Data Extraction

Two reviewers (A.M. and S.M.) independently extracted the demographic and experimental data from the selected studies. When they disagreed, they reviewed the papers together in order to reach joint conclusions. For each study, the following relevant information was extracted and summarized: The characteristics of the sample of subjects under evaluation, the taste tests, and possible neuropsychological comparators; the experimental setting/nutritional intervention of the investigated study group; and the main results of the study with focus on taste changes after the nutritional intervention.

## 3. Results

**Figure 1:**
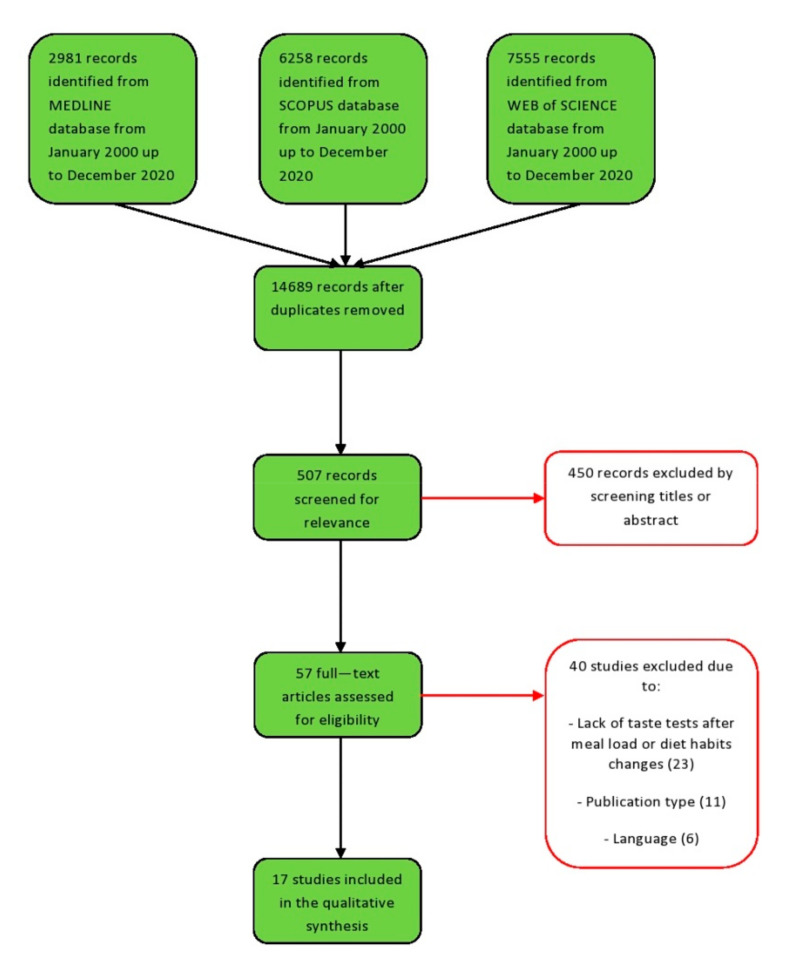
Selection process for the studies included in the scoping review. The Preferred Reporting Items for Systemic Reviews and Meta-Analyses for scoping reviews [PRISMA-sc (according to Tricco et al. [35])] flow diagram depicts the number of records identified, included, and excluded, and the reasons for exclusion, through the different phases of the scoping review.

The literature search retrieved 2981 (Pubmed), 6258 (Scopus), and 7555 (ISI Web of Science) articles evaluating the impact of the nutritional intervention on taste perception. A total of 57 articles were retrieved for full-text review of which 17 were selected and included based on the inclusion criteria in the last 20 years (Figure 1).

The studies’ results are summarized narratively and presented in Table 2 and Table 3, according to the experimental design.

### 3.1. Observational Studies

Five studies were conducted by implementing an observational approach to assess the effect of different nutrients found in free-living dietary habits on the perception of taste: three in healthy subjects [39,40,41] and two in patients [22,42]. The study of Mattes and DiMeglio performed on 50 usual healthy alcohol consumers [39] found that the variance in ethanol and tetralone taste threshold was around twice as high in light users as in heavier users, that a rinse with nonalcoholic beer led to a greater intensity of sweetness compared with other conditions, and higher saltiness and sourness ratings when compared with carbonated water. Bitterness ratings were lower after nonalcoholic beer compared to after carbonated water, and a rinse with beer induced lower bitterness ratings compared with other conditions [39].

Noh et al. stratified 207 healthy participants according to their sodium and zinc intake by means of a dietary intake record method and epithelium sodium channel (αENaC) A663T gene polymorphism (associated with the risk of developing hypertension) [40]. The salty taste threshold was positively correlated with the sodium intake in the whole population but only in women it was significantly lower in the third tertile of total zinc intake and available zinc intake than in the first tertile. A negative correlation between the available zinc intake and salty taste threshold was found in αENaC A663T AA genotype women [40]. Following a similar protocol setting, in a study by Zdilla et al., 363 participants were enrolled, their daily zinc intake was evaluated, and they were tested for zinc taste acuity [41]. Female zinc intake was not correlated with the perception of the taste of zinc sulfate. Male zinc intake was not correlated with zinc taste test scores, but it was significantly correlated with the taste intensity visual analog scale. In 2015, Tucker et al. examined interactions between fat taste (linoleic acid) and dietary fat intake by enrolling 735 subjects, including obese and non-obese subjects [22]. Body fat was not correlated with fat intensity rating and no difference was found in fat intensity rating between obese and non-obese adults except for medium linoleic acid concentration rated higher by lean subjects. In the obese participants, for the medium concentration, mono- and poly-unsaturated fat intake was negatively associated with fat taste intensity ratings [22]. Similarly, 69 Australian females in normal or abnormal (underweight, overweight, or obese) weight conditions were tested by Costanzo et al. for detection thresholds to oleic acid and sensitivities to the five basic tastes (sweet, salty, sour, bitter, and umami) [42]. A 24-h dietary recall was used to assess subjects’ short-term dietary intake. Fat taste sensitivity appeared to be associated with short-term fat intake with the proportion of fat consumed relative to total energy intake rather than the total amount of fat consumed. No significant associations were observed between fatty taste rank and sensitivity to any of the five basic tastes [42].

### 3.2. Interventional Studies

Twelve studies were performed by testing taste after an interventional approach (Table 3): 6 of which enrolled only healthy subjects [43,44,45,46,47,48] and 6 which also included patients affected by different disorders [21,49,50,51,52,53].

In order to reproduce food conditions that possibly lead to taste alterations, some studies were designed to administer a specific food stimulus over a defined period and re-test taste sensitivity. Dalenberg et al. evaluated taste perception in 39 healthy young adults who consumed seven sucralose-sweetened beverages with, but not without a carbohydrate, over 2 weeks [43]. The taste perception was found unaltered while a decreased insulin sensitivity in the healthy participants receiving sucralose combined with maltodextrin was correlated with reductions in the midbrain, and insular and cingulate brain areas were correlated with blood oxygenation level dependent (BOLD)-responses to sweet, but not sour, salty or savory taste as assessed with fMRI. In another short-term study by Noel et al., investigating the impact of 4 weeks of broth administered daily with or without monosodium glutamate (MSG) on suprathreshold taste intensity ratings, liking preferences and ad-libitum test meal, the treatment group showed a marginal difference for the highest aqueous stimuli concentration of umami but not for sweet or salty tastes when compared with the control group and rated the high concentration 5.6 units lower than the baseline [45]. In a longer study (5 months), Wise et al. found that reducing sugar of about 40% in the routinary diet induced participants to rate by the third month low-concentration samples as sweeter than did the control group and to give higher sweetness ratings across a wide range of added-sugar concentrations [47]. Other authors further reduced the time length of nutrients administration or deprivation to evaluate fast taste changes. This is the case of a very short-term protocol, in which the recognition threshold for sweet, sour, salty, and bitter tastants was evaluated immediately before and after the consumption of the same blend of regular or decaffeinated coffee [44]. Interestingly, in both groups of healthy participants the detection threshold for the sweet tastant was increased (*p* < 0.001) while the threshold for the bitter tastant was significantly decreased (*p* < 0.001). However, the decrease of the threshold for the bitter tastant was found to be larger in participants that did not consume coffee daily [44].

Given the interest about the impact of hunger and satiety state on taste threshold, Zverev performed a cross-over interventional, short-term, study in which 16 male participants were tested for taste threshold with a sipping technique for sweet, salty, and bitter qualities in hunger state, after 14–16 h of fasting, and in a satiated state 1 h after a standard dinner or lunch [48]. The recognition thresholds values for the sweet and salty substances were significantly higher during satiety state than in fasting state, while no significant difference was found for the bitter substance in fasting states and that after caloric loading. Following both a similar cross-over protocol and a taste threshold technique, but where the food intake was composed only of a dish of sweetened cream, Pasquet et al. did not confirm the hypothesis of Zverev of an increased sensitivity for sugars and sodium chloride in the fasted state since the within-subjects analysis found no significant threshold shift for all tastants [46].

Given the consequences of some nutrients on people’s health, different studies have been conducted to evaluate the impact of food’s specific components restriction on gustatory and preference in meal choice changes when the study sample is not (or not only) constituted by healthy people but by diseased or elderly subjects. Kusaba et al. conducted a short study comparing salty taste responses among 29 chronic kidney disease (CKD) patients and 11 healthy subjects, before and after 1 week of sodium restriction for the patients [49]. Although both recognition and detection thresholds for salty taste were higher in CKD patients when compared to healthy volunteers at baseline, only the average value of the recognition threshold in CKD patients was found to significantly decrease after 1 week of sodium restriction. In order to investigate whether sodium and potassium intake can lead to altered salty taste responses among adults with high blood pressure, Bolhuis et al. exposed 26 subjects with untreated upper-range prehypertension or stage 1 hypertension to a fully controlled low sodium and low potassium diet for 13 weeks [51]. The results showed that their salty taste responses were not affected over the weeks during the intervention.

Since multifactorial domains have been hypothesized as the reasons for the epidemiological increase in obesity, 20 obese free-living Japanese females, with or without Lys109Arg polymorphism (a common polymoprhism of the leptin receptor gene associated with both insulin and glucose metabolism in women with impaired glucose metabolism and obesity along sweet preference [50]), were enrolled to complete a 12-week weight-loss program, based on energy restrictions through diet and exercise, which aimed at achieving their optimal weight. Before and after the protocol study period, the subjects tasted different concentrations of sucrose [50], and no difference in changes in the sweet taste threshold between the groups (‘with’ versus ‘without’ the Lys109 allele) was found. Serum leptin levels were significantly correlated with the levels of the sweet taste threshold, which decreased significantly in a sucrose solution (*p* = 0.004).

Considering the relationship between obesity and high-fatty food consumption, multiple studies have investigated the possible impact of fatty acid on taste sensitivity. In 2011, Stewart and Keast performed a randomized cross-over dietary intervention on two groups (lean and overweight/obese) of unrestrained eater subjects: one group consumed a high-fat diet followed by a low-fat diet, and the other group consumed a low-fat diet followed by a high-fat diet over a 4-week period [21]. The results showed that the consumption of the low-fat diet increased taste sensitivity to oleic acid (C18:1) among lean and overweight/obese subjects; conversely, the consumption of the high-fat diet significantly decreased taste sensitivity to C18:1 among lean subjects, with no change in sensitivity among overweight/obese persons.

In 2016, Newman et al. conducted more studies on how fat taste sensitivity can be influenced by fat intake: In the first study, 53 overweight/obese participants were randomized and followed either a low-fat diet or a control diet for 6 weeks [52]. In line with the previous study results, the consumption of the low-fat diet significantly decreased the C18:1 threshold (*p* = 0.014), while it had no significant effect on detection thresholds for sucrose (*p* = 0.227) or NaCl (*p* = 0.558). In the second randomized, cross-over study, 32 participants, including 7 overweight/obese individuals, were randomly assigned to one of three different types of breakfast: a high-fat, low-fat or macronutrient balanced frittata [53]. There was no effect of breakfast type on fat taste detection thresholds for either the first or the second testing session of each day.

## 4. Discussion

The main findings of the current scoping review are that nutritional context or dietary habits may induce different degrees of changes on taste tests. The main tastants investigated were fatty acids [21,22,42,52,53], salt [40,49,51], sugar [43,47,50], zinc [41], alcohol [39], and glutamate [45]. Basic tastants (salty, sour, sweet and bitter) evaluation was instead performed in the only two studies evaluating on healthy volunteers the impact of fasting [46,48] and of two different types of coffee [44]. The taste tests investigated, with different techniques and tastants concentrations, the detection threshold [21,39,42,44,46,47,49,50,51,52,53]; the recognition threshold [40,44,48,49]; the intensity rating [22,39,40,41,43,45,47]; and the preference [40,43] (Table 2 and Table 3). Significant changes in these domains were found especially in those studies in which the nutritional intervention, dietary habits or preferences, and interventional approach on the meal load specifically encompassed the tastants included in the taste test [20,21,39,44,51]. This was clearly evident for those studies in which a concordance between the dietary habits or interventional meal load changes and the taste test was present: That is, works investigating the impact of changes in fatty acids levels of diet on detection threshold and intensity rating [21,22,42,52], changes of sodium chloride amount of diet on recognition—but not detection [51]—threshold [40,49], of sweet foods intake on intensity rating [47], and detection threshold [50]. Further, it appeared clear that a specific diet change may not interfere on a taste test devised for different purposes. In the experience of Wise and co-workers [47], sugar restrictions over a 3-month period only induced changes on sweet intensity and pleasantness without impacting the same scales for sodium chloride. Dalenberg et al. [43] demonstrated that the exposure to sweetened beverages did not affect the perception of basic tastants while a decrease insulin sensitivity in the healthy participants receiving sucralose combined with maltodextrin was correlated with reductions in midbrain, insular, and cingulate brain areas with BOLD-responses to sweet, but not sour, salty or savory taste as assessed with fMRI. Beyond theories postulating changes in the expression of heterodimeric taste receptor proteins [54,55], these findings may support that (i) there is a common mechanism that affects peripheral insulin release and the brain response to sweet taste; (ii) peripheral insulin affects the brain response to sweet taste or; (iii) the brain response to sweet taste affects insulin secretion [43]. However, it has been hypothesized that the decrease in insulin sensitivity induces changes in dopamine neuron regions that are important for encoding oral and post-oral reinforcing signals from food [56], which in turn can influence peripheral insulin sensitivity [43,57].

On the other hand, a specific meal load or beverage may considerably affect several tastant thresholds or ratings. This is the case of the study of Fjaeldstad and Fernandes [44] in which the detection threshold for the sweet tastant was found to be higher in a group of subjects regularly consuming coffee while the threshold for the bitter tastant was significantly lower in a group of participants regularly consuming decaffeinated coffee. Similarly, Mattes and DiMeglio [39] found that a rinse with nonalcoholic beer led to a higher sweetness intensity compared with other conditions, and to higher saltiness and sourness ratings when compared with carbonated water and beer. Moreover, the results obtained by Umabiki et al. [50] show how a weight-loss program based on a specific diet and exercise can lead to an improvement in sweet taste, which may be in part accounted for by the decrease in leptin in obese females. Noel et al. [45] came to the same conclusion in an earlier study in relation to the association between increased consumption of umami-rich foods and impaired umami perception in a free-living human population, showing that repeated exposure to umami taste diminishes perceived umami intensity. Curiously, also perceived salt taste tended to decrease across the study period. It is interesting to notice how, mainly in women, the salty taste threshold is found to be affected not only by the deprivation of its stimulus [49] but also by the exposure to umami food [45], as mentioned above, or to zinc intake [40]. This last study suggests that zinc intake plays an important role in determining salty taste acuity and shows how gustatory tests for salty taste work well when salty parameters in the diet are modified or retrospectively studied. Similarly, Kusaba et al. [49] proved that sodium restriction improved the recognition threshold for salty taste in CKD subjects who commonly present taste dysfunction and zinc deficiency. The literature suggests that this internally driven action on salt taste might be regulated by volume or osmosensors in various organs, or by the suppression of hormones associated with salt appetite, or an elevation in cerebrospinal fluid sodium concentrations that has been shown to reduce salt appetite [58]. However, in the study by Bolhuis et al. [51], salty taste tests did not show altered taste responses to sodium and potassium supplementation after a low sodium and low potassium diet. This was hypothesized as possibly due to many biases, including the enrollment of patients with different diseases (e.g., hypertension vs. CKD in Kusaba et al. and Bolhuis et al. study, respectively) and the subsequent administration of various drugs, previously recognized to possibly impact on salty taste threshold [49,59,60,61,62,63].

Collectively, these results are in line with previous literature that suggests that the appetitive tastes of different tastants may be attenuated, or preferences shifted to more intense stimuli with a diet high in the respective taste stimuli [21,64]. These phenomena have been attributed to a down-regulation in expression of specific subunits of sensing G-protein coupled receptors of different nutrients such as umami and fats, which in some cases, given their cross-sensitivity, may also account for a downward trend also for the sensitivity regarding other tastants (e.g., sweet taste) [65,66]. Equivalent associations—suggesting an adaptive relationship that is plastic with either high or low exposure to stimuli—have been reported for diets low in sugar, salt, and especially fat [21,47,67]. Examples of these findings are provided by the interventional studies conducted by Stewart and Keast [21] and Newman et al., 2016 [52,53], which collectively found that taste sensitivity to oleic acid was modulated by exposure to or deprivation of dietary fats. Fat perception decreased in lean subjects following the high-fat diet, while no change was found among overweight/obese subjects [21]. At the same time, in both lean and overweight/obese subjects, significant increases in fat perception were observed following the low-fat diet [21,52], indicating that differences in taste sensitivity to fatty acids may be a result of gustatory adaptation to a high-fat diet and may contribute to excess fat intake because of an attenuated taste response to fatty acids among individuals who habitually consume a high-fat diet [20,52,68]. Interestingly, thresholds for sucrose and NaCl did not change, indicating that the decreases in fat taste threshold were specific to the reduction in fat intake throughout the study period [52]. Further studies implemented these notions and showed that (i) higher fat intakes at the previous eating occasion were significantly associated with decreased intensity ratings [22], (ii) fat taste sensitivity is associated with the proportion of fat consumed with respect to the total energy intake rather than to the total amount of fat consumed [42], and (iii) repeated or long-term—rather than a very short-term—exposure to the tastant could be necessary to elicit a change in detection thresholds [52,53]. Based on similarities in receptor-mediated fatty acid detection within taste receptor cells and enteroendocrine cells of GI tract [67,68], it is suspected that oral fatty acid detection and adaptations to fat exposure would mirror events that occur in the GI tract. Indeed, modulation of fatty acid taste receptor expression has recently been reported in rodents exposed to a high-fat diet [69], elucidating that physical changes in taste receptor expression can be induced by a high-fat diet. Considering all these data, taste tests for fatty acids may help develop personalized diets for obese or overweight people and monitor their daily fat intake with the diet.

Controversial results were obtained about the hypothesis, supporting increased sensitivity to nutrition-related tastants while fasting. Zverev et al. [48] demonstrated that recognition thresholds for sucrose and salt in healthy subjects were significantly lower during caloric deprivation than after caloric loading and that the reactivity of taste to bitter solutions was not affected by food deprivation and satiety. On the other hand, Pasquet et al. [46] failed to demonstrate any statistical difference in taste recognition thresholds between hungry and satiated states. Although these studies were acknowledged to be biased by different factors (such as lack of gender balance and different taste tests methods); open discussions about the reasons for the modulation of taste sensitivity in hungry and satiated states might be summarized as follows: (i) systemic activation of the brain during food motivation or satiety might alter the sensitivity of the central structures involved in the perception of taste stimuli [70]; (ii) the “tuning” efferent influences (mediated through the glossopharyngeal and lingual nerves) on gustatory receptors evoked by hunger or satiety might affect the sensitivity of the gustatory receptors [71]; and (iii) alteration of the autonomic nervous system activity during fasting might contribute to a modulation of the perception of taste stimuli [72,73]. Such findings may be relevant considering the increasing interest around fasting-mimicking diets [74].

In conclusion, the present scoping review reinforces the notions postulating that taste tests might be on one side influenced by the nutritional intervention and that, on the other one, they might be susceptible to a wide span of changes beyond the extent of tastant included in the dietary habits or the interventional meal load changes. This resulted in inhomogeneous findings and in different degrees of findings concordance, depending on the taste test technique, the nutritional intervention, or the dietary changes of the protocol. Indeed, although oral fatty acid tests demonstrated consistent results across the literature, different degrees of results of concordance have been found for other tests investigating tastants’ perception changes such as sodium chloride or sugar. From a speculative point of view, this could depend on various reasons, such as the short duration of the intervention or the random type of meal load, both of which cannot be detected by taste tests; unsuitability of the taste test chosen; and the presence of underlying disorders and different biases in the observational enrolling procedures (body mass index, gender, menstrual cycle) found extensively in literature [64,75,76,77,78,79,80,81]. These aspects, together with individual variability, foster uncertainties in the pathophysiological factors that may collectively be influenced by such variables and finally do not pinpoint sharp borders of taste test reliability in the relative targeted conditions of food habits or changes. Thus, future, more homogeneous, studies are needed to clarify further relationships between taste and nutritional intervention.

## Figures and Tables

**Table 1:** Inclusion criteria for the scoping review summarized according to the Population–Concept–Context (PCC) mnemonic, recommended for scoping reviews [36,37].

**Population**	Healthy and sick adults Any Sex
**Concept**	Taste Tests changes in relation to interventional meal load or observational dietary habits/changes
**Context**	Clinical and non-clinical settings, with focus on chemical sense science, nutrition, and physiological research Original peer-reviewed research articles (cross-sectional and interventional study design), published in English in the last 20 years.

**Table 2:** Observational studies included in the review.

Main Objectives of the Study	Taste Tests	Setting/Protocol and Other Outcome Measures	Subjects	Taste Results	Reference
- To evaluate taste quality for ethanol near threshold. - To evaluate the taste, odor and irritancy thresholds for ethanol within individuals to assess their relative ranking. - To evaluate the effects of rinses with ethanol prior to taste compound sampling.	Taste detection thresholds for ethanol and tetralone using a forced-choice, staircase procedure. Quality ratings (% sweet, % salty, % sour, and % bitter) of ethanol and tetralone were obtained for new samples at threshold and for each of the two concentration steps above each subjects’ threshold concentration. Intensity judgements of suprathreshold concentrations of ethanol and tetralone were obtained by having participants rate concentrations of each in duplicate on 100-cm VAS.	Participants recruited by public advertisement, divided in 17 light beer consumers, 17 casual beer consumers, and 16 regular consumers. Oronasal olfactory detection thresholds for ethanol and trigeminal thresholds in the nasal cavity were performed; anthropometric measurements and diet records were collected.	50 healthy participants (25 females, 25 males; mean age = 28.0 years; mean BMI = 23.6 kg/m^2^)	Taste thresholds for ethanol and tetralone were not significantly different between light, casual, and regular ethanol consumers. The variance in ethanol and tetralone taste threshold was about twice as high in light users compared to heavier users. Suprathreshold intensity ratings of ethanol and tetralone did not differ between ethanol user groups. Rinse with nonalcoholic beer led to higher sweetness intensity (*p* = 0.006) compared with other conditions, and higher saltiness and sourness ratings when compared with carbonated water (*p* = 0.023 and *p* = 0.016 for 0.5% and 1.5% NaCl solutions, respectively) and beer (*p* < 0.001 for both).	Mattes RD and Di Meglio D, 2001 [39]
To evaluate the relationships among αENaC A663T gene polymorphism, zinc status, and salty taste perception including salty taste acuity and preference.	Salty taste acuity determined by measuring the salty taste recognition threshold with 15 sodium chloride test solutions, administered from lowest to highest concentration, and viceversa. Salty taste preference by measuring the preferred concentration of sodium chloride (with a salt-meter) in a clear soup made with soybean sprouts, seasoned with salt until suiting individual taste.	At the first visit, salty taste acuity and preference were measured by sensory evaluation, and dietary intake data were collected using a 24-h recall method. At the second visit, fasting blood samples and anthropometric data were obtained. Additionally, subjects submitted the dietary intake records at the second visit. Three-day dietary record, anthropometric measurements, biochemical assessments, and αENaC A663T genotyping were collected.	207 healthy participants (104 males, mean age = 23.6, mean BMI = 23.0 kg/m^2^; 103 females, mean age = 23.6, mean BMI = 20.9 kg/m^2^)	The salty taste threshold was positively correlated with the sodium intake (r = 0.18, *p* < 0.01). In women, salty taste threshold was significantly lower in the third tertile of total zinc intake and available zinc intake than in the first tertile (*p* = 0.04 and *p* = 0.02). Negative correlation between available zinc intake and salty taste threshold in women (r = −0.21, *p* = 0.04), especially with αENaC A663T AA genotype. In men, salty taste thresholds were similar in all tertiles of available zinc intake.	Noh H et al., 2013 [40]
To evaluate the relationship between the dietary intake of zinc and zinc sulfate taste acuity.	Bryce-Smith and Simpson zinc taste test: Participants were instructed to taste but not swallow 10 mL of a 0.1% zinc sulfate solution and then spit the solution into a sink.	The taste intensity VAS and the zinc-specific food frequency questionnaire calculating zinc intake upon responses to questions regarding intake of foods containing zinc. Participants completed the VAS by marking a line perpendicular to a continuous horizontal line measuring 100 mm at a point that reflected their perceived intensity of the zinc sulfate solution.	363 healthy participants (286 females, mean age = 21.0 years; 77 males, mean age = 20.6 years)	Female zinc intake was not correlated with zinc sulfate taste perception as gauged by both the zinc taste test (r = 0.014, *p* = 0.816) and the taste intensity visual analog scale (r = 0.025, *p* = 0.679). Male zinc intake was not correlated with zinc taste test scores (r = 0.199, *p* = 0.099) but significantly correlated with taste intensity VAS (r = 0.237; *p* = 0.048).	Zdilla MJ et al., 2016 [41]
- To determine if participants could rate the intensity of varying concentrations of linoleic acid presented in the form of edible taste strips - To determine associations between fat taste sensitivity and body fatness, examining how food and beverage intake prior to testing influenced fat taste sensitivity	Participants, wearing nose clips, rated the intensity of edible strips impregnated with either no stimulus (blank) or varying concentrations of linoleic acid on a 100 mm (VAS). The sucralose stimulus served to check that participants could perform the rating task using the VAS scale.	A subset of adults recorded the amount of food and beverage consumed during their last eating occasion prior to testing. One undergraduate dietetics student entered the data into Nutritionist Pro 5.0 dietary analysis software to eliminate inter-rater error. Intensity ratings of an edible taste strip containing sucralose (to check that participants could perform the rating task using the VAS scale), a spicy cinnamon candy (to evaluate trigeminal nerve/chemesthetic sensitivity) and the odor of spearmint extract (to confirm functional olfactory capability) were performed. Anthropometric measurements were collected.	735 (549 adults, 180 children, 6 unknown; 37.9% were male; 85.9% White, 3.1% Asian, 1.4% Black and 8.8% Hispanic; mean age 33; mean body fat percentage among adults 26.9) obese and non-obese participants	There were significant differences (*p* < 0.001) for intensity ratings of low, medium, and high concentrations of fat taste stimuli. Ratings increased in a dose-dependent fashion. There were significant differences (*p* < 0.001) between children’s and adults’ ratings of fat taste intensity on a VAS scale. No differences in fat taste intensity ratings were noted between nonobese (236) and obese adults (304), except for the medium linoleic acid concentration where lean participants rated the taste strip as more intense (*p* = 0.03). Obese and nonobese women were also more sensitive than obese/nonobese men (*p* < 0.001) at the highest concentration. In the obese participants, for the medium concentration, mono- and polyunsaturated fat intake was negatively associated with fat taste intensity ratings (r = −0.21, *p* = 0.021; r = −0.24, *p* = 0.006, respectively).	Tucker RM et al., 2015 [22]
To assess the associations between fat taste thresholds, anthropometric measurements, fat intake, and liking of fatty foods	All participants, wearing nose clips, were tested for detection thresholds to oleic acid (fat taste thresholds) and sensitivities to the five basic tastes (sweet, salty, sour, bitter, and umami). Fatty acid ascending series mixed with long-life fat-free milk by 3-Alternate Forced Choice methodology were used for the fat taste threshold measurement. Ascending concentrations of sucrose, NaCl, citric acid, caffeine and MSG were used for the sensory evaluation of the five basic tastes.	A 24-h dietary recall was used to assess short-term dietary intake (energy intake, total consumption of protein, total fat and saturated, monounsaturated and polyunsaturated fat, carbohydrates, alcohol, and percentage of energy derived from protein, fats, and carbohydrates) using computer software FoodWorks. To evaluate the participants’ ability to discriminate different levels of fat content between food samples, the fat raking task was evaluated. Anthropometric measurements were collected.	69 Australian females (mean age 41.3; mean BMI 26.3) in under- (3), normal- (33), over-weight (13) or obese (20) individuals	Fat taste sensitivity appears to be associated with short-term fat intake, but not body size in this group of females. There was no association between fat taste rank and total dietary fat intake, with or without controlling for energy. This indicates that fat taste sensitivity is associated with the proportion of fat consumed relative to total energy intake rather than the total amount of fat consumed. No significant associations were observed between fat taste rank and sensitivity to any of the five basic tastes (sweet, salty, sour, bitter, and umami).	Costanzo A et al., 2017 [42]

VAS: Visual analogue scale; BMI: Body Mass Index; NaCl: sodium chloride; MSG: monosodium glutamate. *p*: *p*-value; in grey background: studies also involving patients.

**Table 3:** Interventional studies included in the review.

Main Objectives of the Study	Taste Tests	Setting/Protocol and other Outcome Measures	Subjects	Taste Results	Reference
To test if different sweetened beverages may differently impact on taste testing, OGTT, and neuroimaging findings	Before and after the 2-weeks exposure session, participants rated the perceptual qualities of basic tastes (sucrose, 0.56 M; citric acid, 18 mM; NaCl, 0.32 M; quinine, 0.18 mM, and MPG (100 mM) alone and when combined as binary taste mixtures (sucrose-citric acid, sucrose-quinine, sucrose-MPG, citric acid-NaCl and NaCl-quinine). Participants rated the sweetness, sourness, saltiness, bitterness, and umami intensity of each taste using the gLMS. Sweet concentration preference using a sucrose preference test pre- and post-beverage exposure was used. Sweet, sour, salty, umami, and tasteless/odorless stimuli were presented with a custom-designed gustometer in a block design across two fMRI runs.	All groups subjects were randomly assigned to consume for 2 weeks: (1) beverages sweetened with 0.06 g sucralose (sweet uncoupled from calories—LCS), (2) beverages sweetened with 30.38 g sucrose (sweet coupled with calories—Sugar), or (3) beverages sweetened with sucralose and combined with 31.8 g maltodextrin (Combo). GLP-1, insulin, OGTT before and after the exposure session, and anthropometric measurements were recorded.	Tirthy-nine healthy young adults (21 females, 18 males; mean age = 27.8 years; mean BMI = 23.7 kg/m^2^)	Regressing insulin iAUC difference scores on the BOLD-difference maps for sweet taste showed a strong negative relation in several limbic and mesolimbic areas in the Combo group. In this group, the left anterior insula, right middle insula, anterior cingulate, right ventral tegmental area, right putamen, and several cortical areas in the superior temporal gyrus and postcentral gyrus showed a decreased fMRI-BOLD response to sweet taste as a function of iAUC. In a second experiment with maltodextrin consumption alone no insulin sensitivity alteration was seen.	Dalenberg JR et al., 2020 [43]
To investigate the immediate effects of coffee consumption on gustatory and olfactory sensitivity on cohorts of participants reguarly consuming Cofee or Decaffeinated Coffee	Taste-drop-test applied to assess the recognition and detection threshold, consisting of 10 steps of tastant dilutions for sweet (sucrose), sour (citric acid), salty (NaCl), and bitter (quinine) in tap water solvent, with a halving of tastant concentration in every dilution step.	15 min after taste and smell testing, participants were served a lukewarm espresso and instructed to drink it in two sips and to ensure that the coffee was swirled around the entire oral cavity. Within 2 min, participants were provided with 150 mL of tap water and instructed to swirl each sip and drink all of the water. This was done to cleanse their palates prior to retesting smell and taste sensitivity, which took approximately 20 min. Sniffin’ Sticks Test (only in Regular Coffee Group) was performed Same procedure in a group using de-caffeinated coffee	Regular Coffee Group: 101 healthy participants (55 females, 46 males; mean age = 25.5 years). Decaffeinated Coffee Group: 55 healthy participants (30 females, 25 males; mean age = 24.5 years).	Two minutes after coffee consumption, the detection threshold for the sweet tastant was increased (MD = 0.26, *p* < 0.001 and MD = 0.73, *p* < 0.001) while the threshold for the bitter tastant was significantly decreased (MD = −0.56, *p* < 0.001 and MD = −0.64, *p* < 0.001) in Regular Coffee and Decaffeinated Coffee Group, respectively. The decrease in bitter sensitivity (F(1,99) = 4.7975, *p* = 0.031) was found to be larger in participants that did not consume coffee daily (n = 25; decrease in mean bitter sensitivity score of −1.0). A small negative correlation between baseline tastant sensitivity and coffee consumption for all tastants was found: sweet (ρ = −0.17, *p* = 0.09); bitter (ρ = −0.20, *p* = 0.04); salty (ρ = −0.08, *p* = 0.42); sour (ρ = −0.07, *p* = 0.50)	Fjaeldstad AW and Fernandes HM, 2020 [44]
To investigate the influence of a habitual exposure to umami taste on umami taste perception, hedonics, and satiety	Whole mouth suprathreshold taste intensity ratings for aqueous umami, sweet, and salty stimuli were captured on the gLMS. Aqueous taste stimuli were prepared in deionized water and were presented twice, separately, in a series of three ascending concentrations: sucrose for sweet taste at 27.0, 81.0, and 243.0 mmol/L; sodium chloride (NaCl) for salty taste at 11.1, 33.3, and 100.0 mmol/L; MSG for umami taste at 3.0, 9.0, and 27.0 mmol/L.	4-week intervention with one cup of broth daily. The treatment group’s low glutamate vegetable broth (237 mL) was supplemented with 3.8 g MSG. The control group’s broth contained no added MSG, but was sodium-matched with 1.8 g NaCl to ensure both broths contained the same amount of sodium. Both broths contained 15 kcal, 0.3 g fat, 2 g carbohydrates, 1 g protein, and 615 mg sodium. Diet History Questionnaire, anthropometric measurements, Leeds Food Preference Questionnaire, preference of real foods (using an hedonic ratings after consuming samples of different real foods) and an ad-libitum test meal used to assess satiation and satiety (using a VAS before and after both savory and sweet course) were captured at baseline and post-treatment 4-week intervention of testing sessions.	58 healthy participants, 30 in control and 28 in treatment group (72.4% females; mean age = 22.7 years; mean BMI = 21.8 kg/m^2^).	At the start of the intervention, the broth supplemented with MSG tended to be rated as more intensely umami on average compared to the control broth (control: 20.2 ± 2.5; treatment: 27.7 ± 3.2), although not significantly (*p* = 0.06). Treatment group for the highest aqueous stimuli concentration of umami rated the high concentration 5.6 units lower than the baseline. Females in the treatment group but not in control group rated the umami stimulus 8.4 units lower on the gLMS following exposure to MSG. After 4 weeks, the control group increased in consumption of savory foods relative to baseline (42 g), while the treatment group decreased intake (−36 g). Desire for savory foods decreased in the treatment group after 4 weeks	Noel CA et al., 2018 [45]
To test how short-term fasted and satiated states impact on taste thresholds	Determination of taste thresholds carried out in blind conditions, using a series of six pure chemicals in solution in a commercialized drinking water with a staircase-method and presented in a random order with ascending concentrations and up-and-down procedure. Twofold step series (0.3 log-step) included sucrose (2.0 to 1000 mM), fructose (2.0 to 1000 mM), quinine sulphate (0.0004 to 1.6 mM), and purified liquorice (0.015 to 1 g/l of glycyrrhizin), whereas the solutions of NaCl (1.77 to 1000 mM) and PROP (0.001 to 3.2 mM) were diluted with a 0.25 log-step.	Participants were divided in two groups: one group was tested first in the fasted state, whereas the other one was first tested in the satiated state. Both groups were tested twice, within a 2-day interval: in the morning between 8:30 and 10 am, in the fasted state (after an overnight fast), and in the afternoon, about 1 h after a standard lunch, completed before the test, by ad libitum consumption of a standard dish of sweetened cream. Subjective hunger magnitude was recorded on a nine points scale.	24 participants (21 females, 3 males; mean age = 26 years; BMI < 25 kg/m^2^)	No statistically significant variation for recognition threshold of both sugars (sucrose and fructose), purified liquorice, NaCl, PROP, or quinine sulphate when comparing satiated and fasted states. The mean level of subjective hunger differed significantly between the fasted and satiated states (respectively 5.4 ± 2.2 and 1.8 ± 0.9; *p* < 0.0001).	Pasquet P et al., 2006 [46]
To determine how a substantial reduction in dietary intake of simple sugars affects sweetness intensity and pleasantness of sweet foods and beverages	Sweet taste intensity rating by marking 117-mm printed gLMS and pleasantness rating on a 23-point category scale for vanilla puddings and raspberry beverages varied in amounts of added sucrose: 0%, 6.6%, 11%, 25%, 31%, 40%, 47%, and 52% by weight and 0%, 2.5%, 5%, 7.5%, 10%, 12.5%, 16%, 19%, and 25% by weight, respectively. Pleasantness rating on a 23-point category scale for broth and soda crackers varied in amount of sodium chloride: 0.014, 0.06, 01.0, 0.16, 0.25, 0.39, 0.62, and 0.87 mol/L and 0.5%, 1.2%, 2.5%, 4.5%, and 8.5% by weight, respectively. Sensory testing occurred for all concentrations of all stimuli. Sucrose detection thresholds by a forced-choice ascending method of limits. In each trial, subjects sampled a 10-mL aliquot of sucrose solution and two 10-mL water blanks, in random order. Sucrose amounts ranged from 0.0006 to 0.06 mol/L in 12 1.52-fold concentration steps.	After 1 month of normal diet, the control group was instructed to maintain their intake of simple sugars for the first 4 months of the study. The low-sugar group was instructed to lower their sugar intake by 40% (relative to month 1) during months 2–4. Both groups were allowed to follow any diet they wished during the last (fifth) month. Taste tests were performed each month. Anthropometric measurements and food/activity records were recorded.	16 participants in control group (mean age = 34.4 ± 9.7 years; female = 56.3%) and 13 participants in low-sugar diet group (mean age = 36.7 ± 10.2 years; female = 53.4%)	No significant differences between the two groups in rated sweetness intensity during month 1 and 2. By month 3, the low sugar group rated low-concentration samples as sweeter than did the control group (more for pudding than for beverage samples). By month 4, the low-sugar group gave higher sweetness ratings across a wide range of added-sugar concentrations. During month 5, the differences between groups were no longer apparent. In contrast to sweet intensity, there were no significant differences between groups for rated pleasantness. The low-sugar group gave significantly higher sweetness ratings during month 4 than they did during month 1 (*p* < 0.02). No effect on broth and cracker samples.	Wise PM et al., 2015 [47]
To assess the effects of short-term caloric deprivation and satiety on recognition taste thresholds	Recognition thresholds were measured for sweet, salty, and bitter qualities of taste using different concentrations of sucrose (from 1.9 to 233.6 mmol/L), salt (from 1.3 to 171.2 mmol/L), and quinine (from 0.077 to 7.860 mmol/L) solutions, respectively, in cups of 5 mL of distilled water or tested solution with a sipping technique and the standard two-alternative forced-choice technique. Eight concentrations of each substance under test were presented in randomized order. The lowest intensity of a taste stimulus, which could be recognised by taste, was noted as the threshold of recognition.	Participants took their last meal between 6 pm and 7 pm, they missed a breakfast the following morning, and had a lunch at 12.30 pm. All volunteers had the same food at dinner and lunch. Taste thresholds in hunger state in all subjects were measured between 9 am and 10 am, after 14–16 h of fasting. A 1-h interval was allowed between food intake and measurements of taste thresholds in order to avoid the lingering effects of taste adaptation. In eight volunteers, taste thresholds were initially detected in satiated state after a standard dinner and then in hunger state the following morning. In the remaining eight subjects, the order of testing was the opposite: Taste thresholds were initially detected in the morning in hunger state and then in satiated state after a standard lunch. Subjective magnitude of hunger was used to assess at the beginning of the testing procedure on the basis of a self-reported five-points scale.	16 male participants (age = 19–24 years; BMI = 20.5–25 kg/m^2^)	The mean values of recognition thresholds for the sweet and salty substances were significantly higher during satiety state than in fasting state (*p* < 0.05 and *p* < 0.02, respectively). The mean value of recognition thresholds for the bitter substance in fasting states and that after caloric loading did not differ significantly. The difference in taste thresholds between two subgroups of subjects divided on the basis of order of tasting was not statistically significant.	Zverev YP, 2004 [48]
- To analyze the gustatory threshold for salty taste in CKD patients - To investigate the effect of short-term salt restriction on the gustatory threshold	CKD patients and healthy volunteers tasted different concentrations of sodium-impregnated test strips before and after 1 week of protocol study. The impregnated salt concentration was initially 0% and increased in 0.2% intervals from 0.6 to 1.6%. Detection and recognition threshold were assessed.	One week of sodium restriction by means of an educational program including a meal with low salt (5 g/day), low protein (0.8 g/kg/day × ideal body weight) and low potassium (1500 mg/day) was served to all CKD patients. The calories were not altered for either diet, unless the patient had diabetes mellitus. Blood and urinary samples were collected	Group A: 29 patients with chronic kidney disease (CKD; mean age = 62.9 years; 19 males and 10 females; no smoking; 10 with diabetic nephropathy) Group B: 11 healthy volunteers (mean age = 37.7 years; 8 males and 3 females; no smoking and no diabetic nephropathy)	After 1 week of sodium restriction, the average value of the recognition threshold in CKD patients decreased from 0.84 ± 0.27 to 0.76 ± 0.25% (*p* < 0.05) and from 0.68 ± 0.14 to 0.65 ± 0.09% in healthy volunteers (NS). The average value of the detection threshold in CKD patients also decreased from 0.74 ± 0.21 to 0.71 ± 0.23% (NS) and from 0.64 ± 0.08 to 0.62 ± 0.06 in healthy volunteers (NS).	Kusaba T et al., 2009 [49]
- To investigate changes in the sweet taste threshold and leptin serum levels during a weight-loss program. - To determine if the leptin receptor polymorphism (Lys109Arg) affects the taste change.	Subjects tasted 10 different concentrations of sucrose dissolved in sterile water (0.0098, 0.0195, 0.0391, 0.0781, 0.1560, 0.3130, 0.6250, 1.2500, 2.5000 and 5.0000%) according to the whole-mouth gustatory method, before and after 12 weeks protocol study. Detection threshold was measured.	Participants completed a 12-week weight-loss program based on energy restriction through diet and exercise, which aimed at achieving their optimal weight. Changes in serum leptin levels were evaluated during a loss-weight program in connection with a leptin receptor polymorphism (Lys109Arg) that may be related to insulin and glucose metabolism.	20 obese, but otherwise healthy, free-living Japanese females (mean age = 55 ± 7 years; mean weight = 61.7 ± 4.6 Kg; mean BMI = 26.1 ± 1.7 kg/m^2^)	The sweet taste threshold decreased significatively in a solution of sucrose (*p* = 0.004); in contrast, there was no difference in changes to the sweet taste threshold between the groups with versus without the Lys109 allele. Serum leptin levels decreased significantly (*p* = 0.014) and were significatively correlated with those in the sweet taste threshold, independently from the initial threshold levels and the Lys109 allele.	Umabiki M et al., 2010 [50]
To evaluate the effects of a high-fat and low-fat diet on taste sensitivity to oleic acid.	Taste thresholds were established 24 h before initiating a prescribed diet, and again during week 4 of each dietary intervention. Oral sensitivity to C18:1 was determined using a fatty acid ascending series (0.02, 0.06, 1, 1.4, 2, 2.8, 3.8, 5, 6.4, 8, 9.8, and 12 mM) mixed with long-life non-fat milk by a 3-Alternate Forced Choice methodology	All participants were randomized into two groups, which would consume, over a 4-week period, modified fat diets in the following orders: Group 1: high-fat (>45% fat) diet followed by low-fat (<20% fat) diet. Group 2: low-fat (<20% fat) diet followed by high-fat (>45% fat) diet. There was a compulsory 2-week wash-out period between the diets. Fat ranking task was assessed to test the subjects’ ability to discriminate between custards containing small differences (2–4%) in their fat content and to establish what effect consumption of a modified fat diet may have on their ability to detect these differences. Subjects were required to rate their liking and preference of five sets of RF and LF foods by using a nine-point hedonic scale.	19 lean (mean age = 33 years; mean BMI = 23.2 kg/m^2^) and 12 overweight/obese (mean age = 40 years; mean BMI = 28 kg/m^2^) unrestrained eater subjects	Consumption of the low-fat diet increased taste sensitivity to C18:1 among lean and overweight/obese subjects (*p* = 0.05) and increased the subjects’ ability to perceive small differences in the fat content of custard (*p* = 0.05). Consumption of the high-fat diet significantly decreased taste sensitivity to C18:1 among lean subjects (*p* = 0.05).	Stewart JE and Keast RSJ, 2011 [21]
To investigate whether encapsulated sodium and potassium supplementation lead to altered salt taste responses	Participants were subjected to sensory evaluation in week 0, 5, 9, and 13. They started with the detection threshold test by 3-Alternate Forced Choice of NaCl ascending concentrations (0.0125, 0.025, 0.05, 0.1, 0.2, 0.4, 0.8, 1.6, 3.2 and 6.4 g NaCl/L). After that, they rated pleasantness and saltiness intensity of different salt concentrations of water (0, 0.05, 0.16, and 0.5 M NaCl), tomato juice (0.03, 0.05, 0.09, 0.16, 0.28, and 0.5 M NaCl), and bread (0.5%, 1%, 2%, and 4% NaCl)	Participants were exposed to a fully controlled low sodium and low potassium diet (targeted to provide 2 g of sodium and 2 g of potassium at an energy intake of 2500 kcal a day) for 13 weeks. Participants received capsules with sodium (3 g/d), potassium (3 g/d) or placebo, for 4 weeks each, in randomized order in a double-blind crossover design. To compare changes in taste responses and desire-to-eat salty foods, five sweet foods (pancake with sugar, gingerbread, bananas, cookies, and chocolate), and foods that were neither dominant in sweet nor salty taste (‘neutral’ taste) (boiled egg, cucumber, unsalted cashew nuts, rice waffle, yoghurt) were included by using a 100 mm VAS (visual analogue scale). Participants were instructed to collect 24-h urine for the quantitative determination of sodium and potassium.	26 participants (mean age = 66 years; mean BMI = 26.8 kg/m^2^) with untreated upper-range prehypertension or stage 1 hypertension.	The threshold was not affected over the weeks during the intervention diet (*p* = 0.75), not affected by supplementation (*p* = 0.59), and there was no interaction between duration and supplementation (*p* = 0.43).	Bolhuis DP et al., 2015 [51]
To assess the effect of a low-fat or portion control diet on fat taste thresholds, fat perception, and preference	All participants were required to attend one laboratory session at baseline and week 6. They tasted a series of 13 variants of the fatty acid vehicle (non-fat milk) with increasing concentrations of C18:1 (0.02, 0.06, 1, 1.4, 2, 2.8, 3.8, 5, 6.4, 8, 9.8, 12, and 20 mM) to evaluate fat detection threshold, and a series of eight different concentrations of sucrose (0.99, 1.61, 2.75, 4.55, 7.56, 12.62, 21.03, 35.05) and NaCl (2.74, 4.11, 5.82, 8.21, 11.81, 16.77, 23.96, and 34.22 mM) to evaluate sweet and salty detection thresholds by using ascending forced choice triangle tests.	Participants were randomized to follow one of two weight loss diets: (1) consumption of a low-fat (<25% total energy from fat) diet (n = 26) or (2) consumption of a PC (33% total energy from fat, reduction in total energy by 25%) diet (n = 27), for 6 weeks. Anthropometric measurements were recorded. In order to test the subjects’ ability to discriminate between custards containing small differences (2–4%) in their fat content, the fat ranking task was evaluated. Participants completed a preference test with three sets (9-point hedonic scale) of RF and LF foods for the hedonic evaluation.	53 (17 males and 36 females; mean age = 56.5 ± 1.9 years; mean BMI = 32.3 ± 0.7 kg/m^2^) overweight/obese subjects.	Consumption of the LF and PC diets over the 6-week period significantly decreased C18:1 threshold (*p* = 0.014), and the effect tended to be stronger in the LF diet versus PC diet (*p* = 0.060). Diets had no significant effect on detection thresholds for sucrose (*p* = 0.227) or NaCl (*p* = 0.558).	Newman LP et al., 2016 [52]
To determine the effect of a high-fat meal immediately prior to detection threshold testing for oleic acid	Fat taste thresholds to C18:1 (added to long-life skim milk samples at varying concentrations [0.02, 0.06, 1, 1.4, 2, 2.8, 3.8, 5, 6.4, 8, 9.8, and 12 mM]) was determined using triangle tests with ascending forced choice methodology 1 h and 2 h after the frittatas administration. Fat ranking task in which participants were presented with custard samples containing varying amounts of vegetable oil (0, 2, 6 and 10%) and ranking the fattiness of each sample.	Frittatas contained varying amounts of fat: high-fat frittata (60% fat: 21% carbohydrate: 18% protein) contained a total of 9.7 g/100 g of fat and 2.4 g (2410 mg) of C18:1 per 100 g; the balanced-fat frittata (33% fat: 32% carbohydrate: 32% protein) contained a total of 3.0 g/100 g of fat and 1.3 g (1261 mg) C18:1 per 100 g; and the low-fat frittata (20% fat: 43% carbohydrate: 33% protein) contained a total of 1.7 g of fat per serving and 0.7 g (710 mg) C18:1 per 100 g. Hedonic tests using a range of regular-fat and low-fat foods including cream cheese, vanilla yoghurt, and chocolate mousse were used; food consumption record of the day before their first testing session was collected.	32 participants (15 males: mean age = 49.3 years, mean BMI = 24.7 kg/m^2^; 17 females: mean age = 31.5 years, mean BMI = 21.9 kg/m^2^). Out of the total 32 participants, 7 were classified as overweight/obese (5 males, 2 females, mean BMI = 29.1 kg/m^2^)	Fat taste threshold and fat ranking were not different after the three different fat-content breakfasts, as well as no difference in food preferences No significant difference in fatty acid threshold between lean and overweight/obese	Newman LP et al., 2016 [53]

MPG: monopotassium glutamate; NaCl: sodium chloride; gLMS: generalized Labeled Magnitude Scale; fMRI: functional Magnetic Resonance Imaging; BOLD: blood-oxygen-level dependent; GLP-1: glucagon-like peptide 1; OGTT: glucose response to glucose ingestion; iAUC: incremental area under the curve; LCS: low-calorie sweeteners; MD: mean difference; MSG: monosodium glutamate; PROP: 6-n-propylthiouracyl; CKD: chronic kidney disease; C18:1: oleic acid; RF: regular fat; LF: lowered fat; PC: portion control; VAS: visual analogue scale; BMI: body mass index; *p*: *p*-value; NS: not significant; in grey background: studies also involving patients.

## Data Availability

The datasets generated for this study are available on request to the corresponding author.
